# Disparities in the Clinical Encounter: Virginia's African American Children with Special Health Care Needs

**DOI:** 10.5402/2011/273938

**Published:** 2011-09-25

**Authors:** Donald P. Oswald, Joann N. Bodurtha, Janet H. Willis, Donna L. Gilles, Lillian M. Christon, Paula L. Ogston, Susan M. Tlusty

**Affiliations:** ^1^Virginia Commonwealth University, Richmond, VA 23298, USA; ^2^Virginia Department of Health, Richmond, VA 23219, USA

## Abstract

This study analyzed Virginia data from the most recent National Survey of Children with Special Health Care Needs. Logistic regression models were run for six Maternal and Child Health Bureau core outcomes and included demographics, child characteristics, health care providers, and health care access variables as predictors. Race/ethnicity disparities were judged to be present if the race/ethnicity variable was a significant predictor in the final model. Examining the components of disparate outcomes, African American children were found to be less likely than their white counterparts to have a usual source for sick and preventive care and to have a personal doctor or nurse. Their parents were less likely to say that doctors spent enough time, listened carefully, were sensitive to values and customs, and made them feel like a partner. These findings emphasize the need to examine health care disparities at a state level in order to guide efforts at remediation.

## 1. Introduction

The Institute of Medicine (IOM) [1] has fruitfully distinguished between health care difference (in which race/ethnicity groups have divergent absolute values of health care access or outcomes) and disparity (the difference remaining after other potentially mediating factors have been statistically, or otherwise, accounted for). In the United States, disparities have been consistently demonstrated in that racial and ethnic minorities have less access to care, receive poorer quality of care, and are subsequently less satisfied with their healthcare than majority racial/ethnic groups [1, 2]. These disparities are troubling and represent an area of need in terms of research, public health, and public policy.

In particular, healthcare disparities between African Americans and whites have been persistent [3]. Disparities for African Americans have also been noted in other areas including: higher uninsured rates, lower proportions of a usual source of care, and higher unmet prescription needs [4]. Such disparities have been noted for the country as a whole, but there are specific differences for African Americans across states as well. For instance, significant health care disparity patterns between African Americans and whites have been reported in Alabama, Mississippi, and Wisconsin, but not in California, Washington, and Colorado [5]. State-by-state differences illustrate the importance of examining healthcare disparities for particular populations on a state level. In Table 1, selected indicators from Kids Count 2010 [6] show Virginia's data as compared to U.S. overall. Virginia's African American population is over 55% higher than the national percentage, yet the percentage of African Americans living in poverty in Virginia is 22% less than that of the nation. The percentage of Virginia's children with special health care needs (CSHCN) and the percentage of low birth weight and incidence of infant mortality among African Americans are comparable to the U.S. 

Healthcare disparities may result from the interaction of many factors, and there is no easy path to fully understand the causal links among these factors, or to define solutions to the racial disparities that have been observed between different groups in the United States [2]. van Ryn and Fu [7] provide a model of the potential mechanisms through which health and human service providers can have an impact on racial/ethnic disparities. This model includes factors related to help-seeker/patient behavior (e.g., self-disclosure and assertiveness in the medical encounter) and cognitive and affective factors (e.g., attitude, self-efficacy) [7]. Differences in help-seeking behavior have been examined in terms of health care utilization [8]. Potential contributors to disparities for African Americans include perceptions of quality of care, perceived patient-provider relationship, perceptions of health and illness, and perceptions of overall care [9]. 

Provider beliefs and provider behavior in the clinical encounter may also impact racial/ethnic disparities. van Ryn and Fu's [7] model includes factors such as provider beliefs about the help seeker, interpretation of the help seeker's symptoms, and interpersonal behavior in relation to the help seeker. Providers may perceive African Americans and low socioeconomic status patients more negatively on a number of dimensions (e.g., patient intelligence, beliefs about patient's participation in risk behaviors, and expectations regarding patient adherence to medical advice) [7]. Finally, the van Ryn and Fu [7] model also incorporates characteristics of the clinical encounter that might act as obstacles to seeking services and engaging in treatment, exacerbating healthcare disparities. Such factors include problems in communicating with providers, fear of stigma, providers' lack of empathy or understanding of problems, and lack of opportunity to give input in treatment decisions [10]. Conceptual models such as the one proposed by van Ryn and Fu can help to identify potential mechanisms that lead to healthcare disparities for African Americans and provide helpful directions for future research. Research and applied public health work related to understanding and resolving healthcare disparities has been characterized as a moral imperative, and the Healthy People 2010 initiative has a specific focus on resolving racial/ethnic disparities [2].

Current literature documents health care disparities among children with special health care needs (CSHCN) in the U.S. African American children are more likely than white children to have SHCN, as are children from low-income or single-parent households [11] although this finding has not been entirely consistent [12]. Studies exploring family satisfaction with care, the presence of medical home components, and specialty care including dental and mental health provide examples of disparities and contributing factors. In their analysis of the 2001 National Survey of CSHCN, Strickland et al. [13] identified disparities in health care access and medical home for CSHCN in poverty and children of racial and ethnic minority. African American and Latino children were less likely to have a usual source of care or family-centered care. 

Ngui and Flores [14] reported that African American and Latino parents were significantly more likely than white parents to be dissatisfied with care and to report problems with ease of service use. Factors such as minority status, low family income, lack of health insurance, and children with higher functional limitations have been linked to decreased access to and satisfaction with care [12, 14]. Racial/ethnic disparities also exist in unmet needs for specialty, dental, and mental health care services. Factors associated with unmet mental health and dental health care needs for African American and Latino CSHCN included underinsurance and living in poverty [15].

Coker et al. [16] reported significantly lower odds of receiving family-centered care (FCC) for Latino and African American compared with white children even after adjustment for child health, socioeconomic, and access factors. Specifically, disparities were reported for Latino and African American children and children in households with non-English as a primary language with respect to the FCC components of “time spent with the provider” and “sensitivity to the family's values and customs”. Indeed, race/ethnicity disparities in accessing FCC have been reported for all children, not just CSHCN [17]. 

While there is strong support for the presence of race/ethnicity disparities in U.S. health care for CSHCN, there is also good reason to believe that states differ in important ways with respect to the health care system for children and families' experiences in that system [5, 18] and that effective responses to disparities will likewise differ across states. The present study was conducted in collaboration with the Virginia Department of Health; the agency was particularly interested in the question of race/ethnicity disparities in health care for Virginia children. The present study examined the MCHB Core Outcomes and their components, for Virginia children with special health care needs, with particular attention to race/ethnicity disparities. The study was designed to serve as a model for state-level analyses that would inform the development of health care policy that could address race/ethnicity disparities in an intentional fashion.

## 2. Methods

The National Survey of Children with Special Health Care Needs [13] was sponsored by the Maternal and Child Health Bureau (MCHB) and the National Center for Health Statistics. The survey was originally conducted in 2001 and repeated in 2005-2006. The survey sample was constructed to allow for both national- and state-level findings. The project screened 192,083 households for children with special health care needs using the Child and Adolescent Health Measurement Initiative CSHCN screener and completed 40,840 CSHCN interviews, including at least 750 interviews in each state. CSHCN survey data were collected between April 2005 and February 2006. 

The data for CSHCN in Virginia were used as the basis for the present study. Personnel from the Virginia State Department of Health Title V Program collaborated in this effort, guiding the topic selection and contributing to the interpretation of findings. This research was conducted in accordance with prevailing ethical principles.

## 3. Outcome Variables

The primary variables of interest were the six core outcomes for CSHCN identified by the Maternal and Child Health Bureau (MCHB) as reflecting goals for the system of care for this population (see Table 2). CSHCN survey data, along with the algorithm for determining whether the outcomes were met, are available to the public through the National Center for Health statistics web site http://www.cdc.gov/nchs/slaits/cshcn.htm.

## 4. Analysis Plan

The goal of the study was to determine whether there were race/ethnicity disparities in the extent to which Virginia CSHCN met the MCHB outcomes. Logistic regression models were created with “met/did not meet the outcome” as the dependent variable. 

The survey was examined for other variables that might reasonably be expected to have an impact on whether a child would meet the outcomes. Selected predictor variables were divided into four conceptual categories (Demographics, Child Characteristics, Health Care Providers, and Health Care Access).  Table 3 lists the variables included in each category.

Race/ethnicity of the child was included in the Demographics category and was characterized as White/Non-Latino (the reference group), African American/Non-Latino, and Latino. Additional race/ethnicity categories included in the survey were insufficiently represented in the Virginia sample to warrant inclusion as a separate group and were combined in the race/ethnicity category “Other”.

Data analyses were conducted using the *svymean*, *svyprop,* and *svylogit* procedures in the statistical analysis package, Stata 8.1. Use of these procedures allows for the generation of standard errors appropriate to the complex sample design. 

Preliminary models included only race/ethnicity as a predictor and each outcome as the dependent variable. For the primary analyses, four logistic regression models were run for each outcome, one for each category of predictors. Subsequently, significant predictors from each of the four models were combined into a final model for each outcome. Conclusions about significant associations between predictors and outcomes are based on the final models only. Race/ethnicity disparities for each outcome were judged to be present if the race/ethnicity variable was a significant predictor in the final model.

## 5. Results

Analyses were limited to the Virginia CSHCN sample (*N* = 790) which was 58.5% male (SE = .02). The mean age of the sample was 10.0 years (SE = .19). The race distribution was 67.5% White (SE = .02), 25.2% African American (SE = .02), 2.4% Latino (SE = .005), and 4.9% Other (SE = .009). The estimated proportion of children who met each of the outcomes, by race/ethnicity, is provided in Table 4.

Preliminary analyses indicated that African American children, compared to their White counterparts, were significantly less likely to meet Core Outcome 2 (OR = .37; SE = .08; *P* = .000), Core Outcome 3 (OR = .58; SE = 14; *P* = .021), Core Outcome 4 (OR = .42; SE = .10; *P* = .000), and Core Outcome 6 (OR = .33; SE = .14; *P* = .012). These findings are best described as race/ethnicity *differences*; it is not clear from these results whether race/ethnicity is the critical variable determining the differences. Other race/ethnicity groups were not significantly different from Whites for any outcome.

Primary analyses were then run for each outcome, using models that included hypothesized predictors, to examine the data for evidence of *disparities*. Significant predictors in the final model for each of the first three core outcomes are provided in Table 5, along with the associated odds ratio and confidence interval. In each case, African American children were significantly less likely to meet the outcome, compared to White children, taking into account the effects of other variables associated with the outcome; these findings represent *disparities* in health care for CSHCN. For core outcomes 4, 5, and 6, race/ethnicity was not a significant predictor in the final model, and those outcomes were not explored further. 

To clarify the disparities, follow-up analyses were pursued by creating logistic regression models for individual survey items associated with each of the first three outcomes. In each model, race/ethnicity was the predictor, and the individual survey item was the dependent variable.  Table 6 summarizes the results.

These analyses revealed that the parents of African American children were less likely to meet Outcome 1 because they disproportionately fail to “feel like a partner” with their child's physician; they were not less likely to be “very satisfied with services received,” the other component item of Outcome 1.

Component items contributing to the disparity in Outcome 2 (“coordinated ongoing comprehensive care within a medical home”) were more numerous and more varied. African American children were less likely than their white counterparts to have a usual source for sick and preventive care and to have a personal doctor or nurse. In addition, their parents were less likely to say that doctors spent enough time, listened carefully, were sensitive to values and customs, and made them feel like a partner; (parents feeling like a partner with their child's health care provider is a component of both Outcomes 1 and 2). Thus, a variety of health-care-related differences appear to contribute to the race/ethnicity disparity in receiving coordinated ongoing comprehensive care within a medical home.

Under Outcome 3, African American children were less likely to have no gaps in insurance coverage, and their parents were less likely to report that insurance “usually or always meets the child's needs.” These two component items appear to be the primary contributors to the race/ethnicity disparity in having adequate insurance to pay for needed health care services.

Further exploratory analyses examined possible relationships among family-centered care survey items. Each of the family centered care items that showed race/ethnicity differences was positively associated with “feeling like a partner”; if parents reported that the health care provider more often spent enough time (chi-square = 107.78; *P* < 0.001), listened carefully (chi-square = 239.51; *P* < 0.001), or was sensitive to values and customs (chi-square = 180.80; *P* < 0.001), they were more likely to report feeling like a partner. 

Thus, race/ethnicity disparities in family-centered care, an important component of receiving “coordinated ongoing comprehensive care within a medical home,” are associated with differences in feeling like a partner with one's child's health care provider. And “feeling like a partner” appears to be, at least in part, a function of whether the provider spends enough time with the child, listens carefully to parents' concerns, and is sensitive to their values and customs.

## 6. Discussion

Race/ethnicity disparities in health care for children with special health care needs are well documented. The CSHCS Survey provides an opportunity for states to look more closely at those disparities and to consider their implications for remediation. In Virginia, African-American families of CSHCN were less likely than white families to meet MCHB outcomes 1, 2, and 3. 

In the present study, African Americans felt that their CSHCN were less likely to receive ongoing care within a medical home. This discrepancy is in part a function of race/ethnicity differences in health care quality indicators. African American children are much less likely to have a usual source of care and a personal doctor or nurse. 

However, another component of the medical home model relates to the provision of family-centered care. Race/ethnicity differences in family-centered care indicators may help elucidate the medical home disparity. Disparities in Outcomes 1 and 2 and the component differences isolated in the present study point to the importance of investigating how patients and their parents experience the clinical encounter.

Results from the present study are consistent with the Coker et al. finding [16] that Latino and African American CSHCN were significantly less likely to receive family-centered care and that disparities remained after adjustment for child health, socioeconomic, and access factors. Further investigation into the six components of FCC identified disparities with respect to the time spent with the provider and sensitivity to the family's values and customs. Exploratory analyses in the present study suggest that sensitivity to families' values and customs may play an important role in the race/ethnicity differences with respect to whether parents feel like a partner with their child's health care provider.

In Ngui and Flores' analysis of the 2001 National Survey of CSHCN [14], Black and Hispanic parents were significantly more likely than white parents to be dissatisfied with care; however, those differences disappeared after adjusting for family-centered care indicators. Thus, as in the present study, important health care quality indicators were conceptually linked back to differences in the clinical encounter. Similar findings were reported in an analysis of 2000 National Survey of Childhood Health data [19]. 

Three potential mechanisms related to provider attitudes and behavior have been proposed that might produce disparities in health care: bias against minorities, greater clinical uncertainty when interacting with minority patients, and beliefs about the behavior or health of minorities [1]. Each of these mechanisms relates to some aspect of the clinical encounter and may help explain race/ethnicity disparities with respect to family-centered care.

For example, it may be the case that doctors have biases towards African Americans that impact their interactions. Doctors may hold negative beliefs about African Americans behavior or health, of which they may not even be aware, but which might come across in subtle ways such as through nonverbal behavior. Similarly, they may be less likely to collaborate with these patients in making health-related decisions, perhaps in part related to an expectation of lower compliance from African Americans in terms of following through with their recommendations [7]. This process may also be cyclical; African American patients have decreased trust of doctors [20], which may result in doctors being less apt to recommend treatment or engage the patient in treatment planning. Communication is an important factor in the doctor-patient relationship, and doctors have been found to have poorer communication with minority patients. This may be related to discord in certain aspects of communication, such as differences in slang, dialect, and idioms [21]. In any case, poor communication or discordance in communication style may result in perceived racial discrimination contributing to decreased patient satisfaction and involvement in health-related decision making [22, 23]. 

The mechanisms proposed by the IOM [1] offer a convenient framework to guide further research on race/ethnicity disparities. Further exploration of the impact of provider bias, uncertainty, and beliefs on clinical encounters with minority patients will serve to clarify differences in the clinical encounter that produce such disparities.

The present study is limited by the information included in the CHSCN survey. For example, doctor-patient race concordance has been associated with satisfaction with care [24], but provider race/ethnicity information is not available in the data set. The survey also fails to distinguish among type of health care provider (e.g., physician versus nurse practitioner) when reporting on quality indicators including those associated with satisfaction and family-centered care. Disaggregation by type of provider would be useful in identifying targets for intervention. Finally, parents perceptions of care received over the previous 12 months may be affected by recall bias; strongly positive or strongly negative experiences may carry unwarranted weight in perceptions.

Detailed exploration of race/ethnicity disparities in health care for CSHCN may suggest directions with respect to interventions. O'Brien [25] suggested that “relatively little is known about the efficacy of alternative approaches to reducing disparities, or about the strategies that are effective within various racial/ethnic subpopulations” (page 6). O'Brien summarized reports suggesting that

“physician tracking and reminder systems can be effective in improving preventive care and screening services for racial and ethnic minorities, as are initiatives that bypass the physician and give responsibility for offering a service to a nurse or nurse practitioner (e.g. standing orders for adult immunizations). Multifaceted provider interventions may also be effective, but interventions that include only a provider education component are not generally found to be very effective in improving care or narrowing disparities. There is very little evidence yet on the effectiveness of cultural competence training” (page 6).

Pending further investigation of interventions to reduce disparities, providers are left with common-sense responses. A focus on improving the clinical encounter for CSHCN and their families will lead providers to focus on establishing positive interpersonal relations with patients, listening carefully and communicating respectfully, involving parents in decision making, reducing language barriers, and improving ease of use of health care services. Patient education and support interventions may also hold promise such as the *Ask Me 3* program developed by the Partnership for Clear Health Communication for the purpose of improving communication between patients and health care providers.

Findings from the present study emphasize the need to examine health care disparities at the state level in order to guide efforts at remediation. The CSHCN survey is a useful source of data to inform policy makers in their efforts to address race/ethnicity disparities in health care.

## Figures and Tables

**Table 1:** Demographic data and selected child health and poverty indicators for Virginia and US.

Demographic data	US	VA
Nonhispanic white alone	55%	59%
Nonhispanic black alone	14%	22%
Nonhispanic American Indian and Alaskan native alone	1%	0%
Nonhispanic Asian alone	4%	5%
Nonhispanic native Hawaiian and other Pacific Islander alone	0%	0%
Nonhispanic two or more race groups	3%	3%
Hispanic or Latino	22%	11%

Child Health and Poverty Indicators		

Children with special health care needs (All)	19%	21%
Live in poverty (Black/African-American)	36%	28%
Low Birth weight (Black/African-American)	13.4%	12.9%
Infant mortality per 1,000 births (Black/African-American)	13.2	15.4
Confirmed victims of maltreatment (nonhispanic black)	22%	30%

(2010 Kids Count) [6].

**Table 2:** MCHB core outcomes for CSHCN.

(1) Families of children and youth with special health care needs partner in decision making at all levels and are satisfied with the services they receive;
(2) Children and youth with special health care needs receive coordinated ongoing comprehensive care within a medical home;
(3) Families of CSHCN have adequate private and/or public insurance to pay for the services they need;
(4) Children are screened early and continuously for special health care needs;
(5) Community-based services for children and youth with special health care needs are organized so families can use them easily;
(6) Youth with special health care needs receive the services necessary to make transitions to all aspects of adult life, including adult health care, work, and independence.

**Table 3:** Predictor variables from conceptual categories.

Included from demographic models:
(i) race/ethnicity
(ii) age
(iii) sex
(iv) metropolitan statistical area (MSA) status (i.e., in MSA/not in MSA)
(v) household income (% of poverty level)
(vi) highest level of education of anyone in the household
(vii) whether the child was uninsured (yes/no)*
(viii) whether primary language spoken in household is English (yes/no)*
(ix) family structure
(x) whether the child's health care has caused financial problems (yes/no)

Included from child characteristics models:

(i) stability of the child's health care needs
(ii) whether the child has emotional problems (yes/no)
(iii) whether the child has behavioral problems (yes/no)
(iv) severity of the child's condition or problem
(v) whether the child receives special education (yes/no)

Included from healthcare provider models:

(i) child has health care source (yes/no)*
(ii) child has usual routine preventive care source (yes/no)*
(iii) child has a personal doctor or nurse (yes/no)*
(iv) number of doctor visits in the past 12 months
(v) number of ER visits in the past 12 months

Included from healthcare access models:

Child's health care delayed/foregone in the past 12 months (yes/no)
(ii) Child received all needed preventive dental care including checkups (yes/no)
(iii) Child received all needed prescription medicines (yes/no)

*Omitted for some outcomes because the variable was included in the definition of the outcome or because of low cell size.

**Table 4:** Estimated proportion (SE) meeting outcomes by race/ethnicity.

Outcome	Racial/ethnic group			
	White	African American	Hispanic/Latino	Other
(1) Families of children and youth with special health care needs partner in decision making at all levels and are satisfied with the services they receive	.62 (.02)	.53 (.05)	.64 (.10)	.52 (.10)
(2) Children and youth with special health care needs receive coordinated ongoing comprehensive care within a medical home	.49 (.02)	.26 (.04)	.39 (.10)	.34 (.09)
(3) Families of CSHCN have adequate private and/or public insurance to pay for the services they need	.67 (.02)	.55 (.05)	.80 (.08)	.58 (.09)
(4) Children are screened early and continuously for special health care needs	.70 (.02)	.49 (.05)	.59 (.11)	.72 (.08)
(5) Community-based services for children and youth with special health care needs are organized so families can use them easily	.90 (.01)	.87 (.04)	.82 (.10)	.92 (.05)
(6) Youth with special health care needs receive the services necessary to make transitions to all aspects of adult life, including adult health care, work, and independence	.43 (.03)	.20 (.07)	.42 (.16)	.44 (.15)

**Table 5:** Significant predictors for core outcomes.

Predictor	Odds ratio	*P*	95% confidence interval
Outcome (1): families partner in decision making and are satisfied			

Being African American	.57	.023	.35–.93
Living in a two-parent stepfamily	2.23	.012	1.19–4.18
Living in some other family configuration^1^	.34	.035	.12–.93
Child's care caused financial problems	.40	.000	.25–.64
Child has emotional problems	.37	.000	.22–.64
Child's care delayed/foregone in last 12 months	.27	.007	.10–.69

Outcome (2): coordinated ongoing comprehensive care within a medical home			

Being African American	.39	.000	.23–.65
Being Other race/ethnicity^2^	.39	.043	.15–.97
Being female	.66	.022	.46–.94
Living in some other family configuration	.29	.036	.09–.92
Child has emotional problems	.44	.005	.25–.78
Child has behavior problems	.54	.019	.32–.90
Increased number of ER visits	.86	.046	.74–.99

Outcome (3): adequate private or public health insurance			

Being African American	.60	.029	.38–.95
Child's care caused financial problems	.22	.000	.14–.36
Child's care delayed/foregone in last 12 months	.12	.000	.05–.29

^1^Other family configuration meant: not (a) two parent biological/adopted, (b) two parent stepfamily, or (c) single mother, no father present.

^2^Other race/ethnicity meant: not (a) White, (b) African American, or (c) Hispanic/Latino.

**Table 6:** African American differences on individual survey items.

Outcome/item	Odds ratio	*P*	95% CI
(1) (a) Providers usually or always make the family feel like a partner	.36	0.001	.19–.67
(b) Family is very satisfied with services received	.68	NS	

(2) (a) The child has a usual source for sick care	.17	0.000	.07–.45
(b) The child has a usual source for preventive care	.13	0.001	.04–.45
(c) The child has a personal doctor or nurse	.15	0.000	.06–.34
(d) The child has no problems obtaining referrals when needed	1.38	NS	
(e) The child receives effective care coordination			
(i) Family is very satisfied with doctors' communication with each other	.87	NS	
(ii) Family is very satisfied with doctors' communication with other programs	1.02	NS	
(iii) Family usually or always gets sufficient help coordinating care, if needed	.85	NS	
(f) The child receives family-centered care			
(i) Providers usually or always spend enough time	.36	0.000	.21–.62
(ii) Providers usually or always listen carefully	.36	0.001	.19–.66
(iii) Providers are usually or always sensitive to values and customs	.26	0.000	14–.47
(iv) Providers usually or always provide needed information	.66	NS	
(v) Providers usually or always make the family feel like a partner	.36	0.001	.19–.67
(vi) An interpreter is usually or always available when needed	Unable to calculate		

(3) (a) The child has public or private insurance at time of interview	.36	NS	
(b) The child has no gaps in coverage during the year before the interview	.35	0.008	.16–.76
(c) Insurance usually or always meets the child's needs	.39	0.006	.20–.76
(d) Costs not covered by insurance are usually or always reasonable	.79	NS	
(e) Insurance usually or always permits child to see needed providers	.54	NS	
